# Calmodulin Domain Protein Kinase PiCDPK1 Regulates Pollen Tube Growth Polarity through Interaction with RhoGDI

**DOI:** 10.3390/plants11030254

**Published:** 2022-01-19

**Authors:** Nolan Scheible, Gyeong Mee Yoon, Andrew G. McCubbin

**Affiliations:** School of Biological Sciences and Center for Reproductive Biology, Washington State University, Pullman, WA 99164, USA; nolan.scheible@wsu.edu (N.S.); yoong@purdue.edu (G.M.Y.)

**Keywords:** pollen, tip growth, calcium, calcium dependent protein kinase, Rho Guanine Dissociation Inhibitor, ROP GTPase, RhoGDI displacement factor, polarity

## Abstract

The pollen-specific calcium-dependent protein kinase PiCDPK1 of *Petunia inflata* has previously been shown to regulate polarity in tip growth in pollen tubes. Here we report the identification of a Rho Guanine Dissociation Inhibitor (PiRhoGDI1) as a PiCDPK1 interacting protein. We demonstrate that PiRhoGDI1 and PiCDPK1 interact in a yeast 2-hybrid assay, as well as in an in vitro pull-down assay, and that PiRhoGDI1 is phosphorylated by PiCDPK1 in vitro. We further demonstrate the PiRhoGDI1 is capable of rescuing the loss of growth polarity phenotype caused by over-expressing PiCDPK1 in vivo using stable transgenic plants. We confirmed that PiRhoGDI1 interacts with a pollen-expressed ROP GTPase isoform consistent with the established role of RhoGDIs in negatively regulating GTPases through their membrane removal and locking them in an inactive cytosolic complex. ROP is a central regulator of polarity in tip growth, upstream of Ca^2+^, and PiCDPK1 over-expression has been previously reported to lead to dramatic elevation of cytosolic Ca^2+^ through a positive feedback loop. The discovery that PiCDPK1 impacts ROP regulation via PiRhoGDI1 suggests that PiCDPK1 acts as RhoGDI displacement factor and leads us to propose a model which we hypothesize regulates the rapid recycling of ROP GTPase at the pollen tube tip.

## 1. Introduction

Highly polarized growth is a characteristic of a number of specialized eukaryotic cell types including animal neurons, fungal hyphae, and higher plant pollen tubes and root hairs. These cells grow by a process known as “tip growth” in which expansion/extension is continuously restricted to an apical domain [1]. Pollen tubes extend to form a conduit through which sperm cells are transported through female floral tissues to the ovules, and thus are critical to sexual reproduction in higher plants. In addition to their biological significance, they provide a tractable model with which to study tip growth. Though pollen is multicellular, it consists of relatively inactive sperm cells located within a highly active vegetative cell that grows to form a pollen tube, providing an opportunity to investigate polar growth at the cellular level with relative ease.

Ca^2+^ has long been known to play a pivotal role in regulating pollen tube growth [2,3,4,5,6]. Growing tubes exhibit a steep-tip-focused free Ca^2+^ gradient, the perturbance of which results in reversible cessation of tip growth [3,7,8]. Calcium dependent protein kinases, particularly Calmodulin-domain-like protein kinases (CDPKs) have been implicated as being one of the immediate sensors and response elements to this Ca^2+^ signal, their kinase activity being activated by elevated Ca^2+^ levels leading to phosphorylation of downstream components of these signaling pathways [9]. Expression profiling data indicate that 16 CDPK isoforms are expressed in pollen in *Arabidopsis thaliana* and knock-out mutants of 6 of the 13 tested exhibit mutant phenotypes in pollen germination and or tube growth [9]. Over-expression of *Petunia inflata CDPK1* (PiCDPK1) in pollen causes depolarized growth, whereas expression of a constitutively active mutant version of this isoform severely inhibits growth [10]. An analysis of a double-knock out of the functionally redundant isoforms of *A. thaliana CPKs-17* and *-34* (the At*CPK* isoforms most closely related to PiCDPK1) provided genetic corroboration of the biological and broader phylogenetic relevance of these results. Double T-DNA insertion mutants of *AtCPK-17* and *-34* exhibited a ~3 fold reduction in pollen tube growth rate and 350 fold reduction in transmission efficiency [11]. Identification of the downstream targets of protein kinases is key to understanding their biological function. AtCPK34 was recently implicated in modulating the activity of two pollen-specific aquaporins, NIP4-1 and NIP4-2, through phosphorylation of the Serine-267 residue [12]. Of the other *A. thaliana* CPK isoforms that are expressed in pollen, reports suggest that *AtCPK2* and *20* participate in regulating anion concentrations in pollen tubes by phosphorylating and thereby mediating the activity of the anionic membrane channel SLAH [13]. AtCPK11 and 24, with the former capable of phosphorylating the latter, participate in regulating the inward movement of potassium through the channel SPIK1 [9]. AtCPK32 interacts with and phosphorylates a cyclic nucleotide-gated calcium channel and is key for maintenance of the tip-focused calcium gradient [14]. Though the immediate downstream targets of PiCDPK1 in tip growth have not been identified, over-expression of PiCDPK1 causes a dramatic elevation of Ca^2+^ at the pollen tube tip, which as it is Ca^2+^-activated, suggests the possibility that it may be involved in a positive feedback loop [10]. Combined these studies suggest that CPK gene family members and their interactions with downstream effectors are critical components of Ca^2+^ signaling pathways in pollen tube growth.

Signaling upstream of Ca^2+^ involves the action of a class of plant-specific Rho GTPases, designated ROPs (Rho-like GTPase of Plants) [15,16,17,18]. There is substantial evidence that ROP GTPases are central regulators of tip growth in pollen tubes [15,16,17,18] and that they regulate both the tip-focused Ca^2+^ gradient and apical actin cytoskeleton through antagonistic effector pathways [19,20]. Specifically, ROPs mediate, through the actions of immediate downstream effectors, F-actin organization so that actin is present in bundles at the tube, as opposed to the cables present further back in the tube [20,21]. Plasma membrane localization is essential to ROP biological function and is mediated by isoprenylation. Spatial restriction of the biological activity of ROP is also critical to tip-growth, and in common with other GTPases, is regulated by a number of classes of regulatory protein that mediate alternation between GTP-bound biologically active and GDP-bound inactive states, as well as membrane association. Rho guanine exchange factors (RhoGEFs) positively regulate ROP activity and are themselves positively regulated by pollen receptor-like kinases (PRK) [22]). The localization of RhoGEFs is important for spatially restricting activation of ROPs at the tube tip [23,24].

Negative regulation of ROP occurs by stimulation of the intrinsic GTPase activity of these proteins (causing hydrolysis of bound GTP to GDP) by Rho GTPase activating proteins (RhoGAPs), and secondarily by the removal of ROP from the plasma membrane by Rho guanine dissociation inhibitors (RhoGDIs). In pollen, RhoGAPs activate ROP GTPases activity in the pollen tube shank behind the tip, confining biological activity to the tube apex [25]. A RhoGAP spatially coordinates the activity of ROP, acting as a rheostat for ROP signaling in times of increased or decreased expansion [24]. RhoGDI binds preferentially to GDP bound ROP behind the tip, causing its dissociation from the plasma membrane and sequestration in the cytosol [26]. Interestingly, recycling of ROP from its complex with RhoGDI appears to be critical to pollen tube growth, as mutant RhoGDI that binds but cannot release ROP inhibits growth [26]. RhoGDI function has also been implicated in regulating the distribution of cell wall components in pollen tubes, as a triple knockout mutant in *A. thaliana* exhibits an increase in cell wall components at the tube tip, as well as altered ROP activation [27].

Recycling and activation of ROP requires the action of RhoGDI displacement factors (RhoGDFs) that promote dissociation of ROP from RhoGDI and its re-association with the plasma membrane. RhoGDFs have not yet been identified in plants but it has been speculated that PtdIns(4,5) *P_2_*, which possesses RhoGDF activity in animal cells, may fulfill this function [16,28]. However, this is unlikely to be the sole RhoGDF activity, as in animal cells phosphorylation of Rho GTPase affects its affinity for binding to RhoGDI [29], in addition post-translational modifications of RhoGDI, including phosphorylation by kinases including protein kinase C [30] and p21-activated kinase also influence dissociation of RhoGDI-Rho complexes [31]. Subsequent to plasma membrane recruitment, the biological activity of ROP is activated by Rho guanine exchange factors (RhoGEFs), which promote exchange of the GDP bound by ROP for GTP, and a novel class of plant-specific RhoGEFs has been identified [19,32]. In pollen tubes, RhoGEF interacts with a pollen receptor kinase (PRK) in a manner that leads to the recruitment of RhoGEF to the plasma membrane at the tube apex as well as the release of an intrinsic C-terminal inhibition of RhoGEF activity [32,33]. Three distinct RhoGEFs from *Arabidopsis* interact with PRKs, in some cases forming protein complexes, and these interactions are essential for tube elongation and seed set. The interaction between PRK and RhoGEF leads to an increase in ROP activity, suggesting that RhoGEF becomes activated by this interaction [22,34]. Significantly, this direct link between ROP pathways and a transmembrane receptor provides a mechanism by which pollen tubes may perceive extracellular signals and orient polar growth in response.

We previously reported that the pollen-specific CDPK isoform PiCDPK1 plays a pivotal role in regulating pollen tube growth polarity [10]. As a protein kinase directly regulated by Ca^2+^, PiCDPK1 is necessarily an effector of this secondary messenger in pollen tubes. How PiCDPK1 activity mediates tip growth is unclear and identification of PiCDPK1 substrates is an important goal in elucidating these processes. Here we report the identification of a RhoGDI from pollen of *Petunia inflata* (designated PiRhoGDI1) as a downstream target of PiCDPK1 using the yeast 2-hybrid system. Several lines of evidence supporting this interaction are presented including in vitro pull-down and phosphorylation assays, and an in vivo rescue of the loss-of-polarity induced by over-expressing PiCDPK1 by co-over-expressing PiRhoGDI1 in pollen tubes of stable transgenic plants of *Nicotiana tabacum*.

## 2. Results

### 2.1. Yeast 2-Hybrid Library Screening

**Figure 1:**
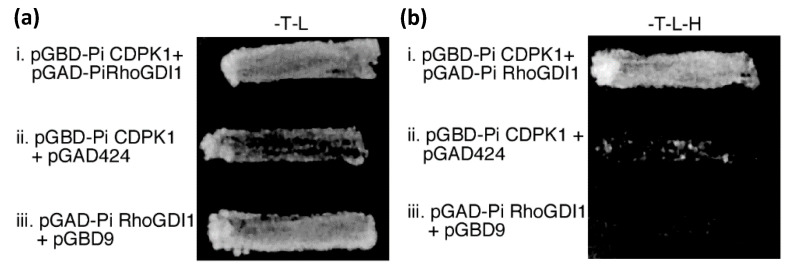
PiRhoGDI1 interacts with PiCDPK1 in vivo. Yeast transformed with bait (pGBD) and prey (pGAD) constructs or empty vectors (as labeled) were grown on (**a**) media lacking tryptophan and leucine (plasmid selection), and (**b**) media lacking trytophan, leucine, and histidine, providing selection for both plasmids and interaction of fusion proteins. Only cells transformed with pGBD-PiCDPK1 and pGAD-PiRhoGDI1 grew well on histidine selection, suggesting these clones encode proteins that interact.

In an effort to identify substrates, a pollen tube (yeast 2-hybrid) cDNA library was screened with a bait construct encoding the N-terminal variable and kinase domains of PiCDPK1. DNA sequencing revealed that one class of five independent positive clones identified represented a cDNA encoding a full-length homolog of *AtRhoGDI1* (At3g07880, BLASTP E value = 7 × 10^−42^). This cDNA possessed an in-frame 5′ stop codon that theoretically prevented synthesis of a fusion protein with the yeast GAL4 activation domain, however as RhoGDIs regulate ROP GTPases, which, similar to PiCDPK1, have been shown to regulate pollen tube growth polarity [10,15,16,17,18], we chose to investigate this class further, naming the gene *P. inflata RhoGDI1* (*PiRhoGDI1*) (GenBank acc. # DQ905960). The coding region of PiRhoGDI1 was re-cloned in the yeast 2-hybrid prey vector to create an in-frame fusion with the GAL4 activation domain. The resulting construct was determined to facilitate yeast growth under histidine selection in the interaction assay when co-transformed with the in-frame PiCDPK1 bait construct (Figure 1a), but neither prey nor bait construct conferred this ability when co-transformed into yeast with the respective empty partner vectors (Figure 1b). These results suggested that PiRhoGDI1 did interact with PiCDPK1 and that neither protein was capable of activating the GAL4 promoter alone. Whether the initial identification of this gene was entirely fortuitous is unclear, an alternative possibility being that the 5′ stop codon in the cDNA of the initial clone identified was removed during RNA processing in yeast. We next sought to confirm, and investigate the nature of, the interaction between PiCDPK1 and PiRhoGDI1 using alternative methodologies.

### 2.2. PiRhoGDI1 Interacts with and Is Phosphorylated by PiCDPK1 In Vitro

**Figure 2:**
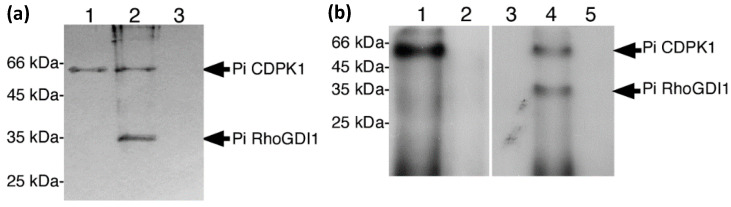
PiCDPK1 interacts with and phosphorylates PiRhoGDI1 in vitro. (**a**) Pull down assay. His-tagged protein samples were incubated with phenyl sepharose resin, washed, separated by SDS-PAGE, and blotted to PVDF membrane. Immuno-detection was performed with anti-His tag monoclonal antibody. Lanes represent the following samples: (1) PiCDPK1 alone, (2) PiCDPK1 and PiRhoGDI1, (3) PiRhoGDI1 alone. PiRhoGDI1 was only detected in combination with PiCDPK1, suggesting that the two proteins interact. (**b**) In vitro phosphorylation assay. Phosphorylation assays were performed in the presence of 50 µM Ca^2+^ to stimulate PiCDPK1 or 1 mM EGTA to inhibit PiCDPK1. Samples were separated by SDS-PAGE and subjected to autoradiography. Lanes: (1) PiCDPK1 + Ca^2+^, (2) PiCDPK1 + EGTA, (3) PiRhoGDI1 + Ca^2+^, (4) PiRhoGDI1 + PiCDPK1 + Ca^2+^, (5) PiRhoGDI1 + EGTA. PiRhoGDI1 was phosphorylated only in the presence of PiCDPK1 and Ca^2+^.

In the presence of Ca^2+^, the calmodulin-like domain of CDPKs binds specifically to phenyl sepharose by hydrophobic interaction [35]. Exploiting this characteristic, we designed an assay using phenyl sepharose resin to selectively pull-down PiCDPK1 and any interacting protein. We employed recombinant His-tagged PiCDPK1 and PiRhoGDI1, allowing detection of both proteins on a single protein blot using an anti-His tag monoclonal antibody. As shown in Figure 2a, PiCDPK1 bound to phenyl sepharose as expected and PiRhoGDI1 was pulled-down only in the presence of PiCDPK1 providing a second line of evidence of protein–protein interaction between PiRhoGDI1 and PiCDPK1.

As physical interaction between a protein with a protein kinase does not necessarily infer phosphorylation, we also assessed whether PiRhoGDI1 was substrate of PiCDPK1 in vitro. Phosphorylation assays were performed in the presence of P^32^ labelled ATP, 50 µM Ca^2+^ or the Ca^2+^ chelator EGTA (1 mM) and with or without addition of PiCDPK1, and the products of reactions analyzed by SDS-PAGE and autoradiography. PiCDPK1 exhibited autophosphorylation only in the presence of Ca^2+^ as expected, and PiRhoGDI1 was found to be phosphorylated in the presence of Ca^2+^ and PiCDPK1, but not in the absence of PiCDPK1 or the presence of 1 mM EGTA and PiCDPK1 (Figure 2b). These results are consistent with PiRhoGDI1 being a PiCDPK1 substrate in vitro and the phosphorylation observed not being caused by a contaminating protein kinase. Using Michaelis–Menton conditions, we then investigated the kinetics of this reaction and determined an approximate K_m_ of 2.2 µM and a V_max_ of 1920 pmol min^−1^ mg^−1^, the latter being similar to the V_max_ of PiCDPK1 phosphorylation of the synthetic CDPK substrate syntide-2 (2042 pmol min^−1^ mg^−1^) [10].

### 2.3. Confirmation of the Interaction of PiRhoGDI1 with ROP GTPase

RNA blot analysis indicated that PiRhoGDI1 is predominantly expressed in pollen, being first detectable in anthers from 15–20 mm buds (corresponding to pollen mitosis I), peaking in mature pollen and remaining high in pollen tubes (Figure S1), this expression pattern mimics that of PiCDPK1 [10]. Expression in other tissues was not detected but cannot be excluded in under-represented cell types, notably root hairs, which also grow by tip growth. A RhoGDI isoform from tobacco (Nt*RhoGDI2*) has been demonstrated to interact with ROP GTPase and suppress loss of growth polarity caused by *ROP* over-expression [26]. To confirm that PiRhoGDI1 also interacts with ROP, we cloned a *ROP* GTPase isoform from *P. inflata* pollen (Pi*Rop1*; GenBank acc. # DQ905959), and determined that it exhibited 94% amino acid identity with At*Rop1* (At3g51300). PiRop1 was then cloned into a yeast 2 hybrid vector and the interaction between PiRhoGDI1 and PiRop1 confirmed using the two genes in prey and bait constructs (respectively) in a yeast 2-hybrid interaction assay (Figure S2).

### 2.4. PiCDPK1 Suppresses the Effect of PiRhoGDI1 Over-Expression In Vivo

RhoGDIs are negative regulators of Rho class GTPases, acting to lock them in a biologically inactive state in cytosolic complexes. RhoGDIs are themselves regulated by RhoGDI-displacement factors (RhoGDFs), which promote the re-association of the GTPases with the plasma membrane, making them accessible for biological re-activation by RhoGEFs [36]. The molecular identities of RhoGDFs are not well understood in plants. Of particular relevance to this study, however, phosphorylation of RhoGDI by kinases is involved in fast recycling of Rho GTPases in animal cells [29]. For example, RhoGDI phosphorylation by protein kinase C [30] and p21-activated kinase [31] stimulates release of the GTPase from the inhibitor complex. This precedent led us to hypothesize that phosphorylation of PiRhoGDI1 by PiCDPK1 may regulate release of ROP GTPase from the RhoGDI complex in pollen tubes and design an experiment to test this hypothesis.

### 2.5. Co-Expression with PiRhoGDI1 Rescues the PiCDPK1 Overexpression Phenotype

As noted above, over-expression of PiCDPK1 in pollen and pollen tubes causes a loss in growth polarity (ballooning). We reasoned that if PiCDPK1 negatively regulates RhoGDI, over-expressing the two proteins together should at least partially neutralize the effect of excess PiCDPK1 by both titrating kinase activity away from endogenous RhoGDI and by increasing the non-phosphorylated pool of RhoGDI. We chose to use stable transformants to investigate this possibility to facilitate accurate quantification of expression the transgenes. *Nicotiana tabacum* was used for these transgenic experiments to take advantage of its ease of transformation, suitability for pollen tube growth and microscopy assays (large pollen), whilst retaining close phylogenetic proximity to *Petunia*. The strong pollen-specific *Lat52* promoter [37] was chosen to drive the constructs as this promoter was previously used to drive expression of PiCDPK1 in transient pollen transformation assays [10].

**Figure 3:**
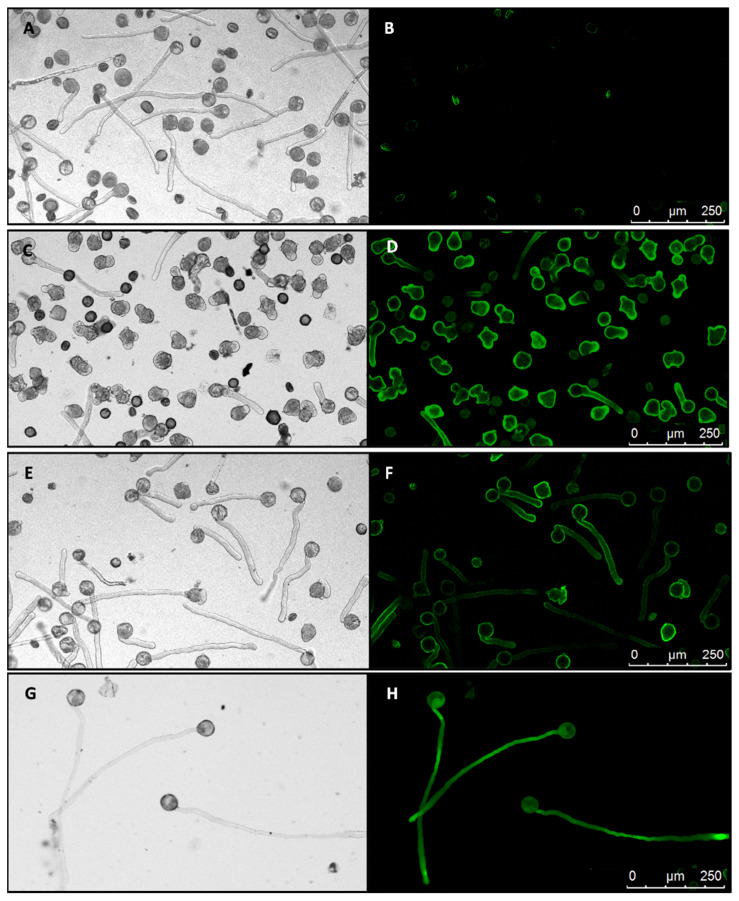
Effect of expressing PiCDPK1 alone and with PiRhoGDI1. Images show pollen cultured in vitro for 4 h at 28–30 °C. Paired images show light micrographs and equivalent fluorescence micrographs (black background). (**A**,**B**), wild type pollen tubes, the slight fluorescence in (**B**) is associated with a low percentage of dead pollen which auto-fluoresces. (**C**,**D**), pollen tubes from line OE1 (homozygous for *Lat52-PiCDPK1-GFP* alone) exhibiting loss of growth polarity (OE1). (**E**,**F**), pollen tubes from line Rescue 2 (homozygous for both *Lat52-PiCDPK1-GFP* and *Lat52-PiRhoGDI1*), these tubes did not lose polarity and resemble wild type pollen tubes. (**G**,**H**) from a homozygous p*Lat52-GFP* transgenic line showing pollen tubes expressing GFP alone.

The goal of this experiment was to assay the ability of PiRhoGDI1 to rescue the PiCDPK1 over-expression phenotype in pollen tubes. To interpret the results of this experiment it was necessary to be able to accurately assess expression levels of the transgenes. As pollen are haploid it was necessary to generate lines that were homozygous for the transgenes to provide populations of pollen with uniform genotype. Further it was important that rescue lines expressing both transgenes expressed similar expression levels of PiCDPK1 to those expressing PiCDPK1 alone, to improve our chances of achieving this we employed sequential transformations. We first used *Agrobacterium*-mediated transformation of leaf strips to generate stable tobacco lines transformed with *pLat52-PiCDPK1:GFP*. Pollen of these lines was cultured in vitro and those that exhibited GFP fluorescence in 50% of the pollen (indicating a T-DNA single insertion) identified. The transformed pollen also recapitulated previously reported the loss-of-polarity phenotype [10]. Selected T0 plants were then self-pollinated, and the resultant seed was used to generate T1 progeny. T1 plants homozygous for the transgene were then identified based on 100% of the pollen exhibiting GFP fluorescence and at least some loss-of-polarity (Figure 3C,D). Such plants were self-pollinated and some of the resultant seed was used to grow a T2 population of seedlings homozygous for the T-DNA. In a similar manner transgenic plants homozygous for *pLat52-GFP* were also generated to control for potential effects of expressing high levels of GFP on pollen tube growth (Figure 3G,H). T2 homozygous *pLat52-PiCDPK1:GFP* seedlings were then used as the leaf material for a second round of *Agrobacterium*-mediated transformation with *pLat52-PiRhoGDI1* to generate rescue lines. Primary transformants from this second round were assessed for presence of the *pLat52-PiRhoGDI1* transgene by RT-PCR of pollen cDNA and in vitro pollen tube growth phenotypes assessed.

Transformants expressing PiRhoGDI1 were found to bear pollen 50% of which exhibited loss of polarity and 50% grew in a polar manner, but 100% exhibited PiCDPK1:GFP fluorescence consistent with the *pLat52-PiRhoGDI1* transgene being capable of rescuing loss of polarity caused by PiCDPK1. These plants were self-pollinated, and the resulting seed used to generate a T1 population. From this population, lines were then identified for which 100% of the pollen exhibited both PiCDPK1:GFP fluorescence (indicating homozygosity for *pLat52-PiCDPK1:GFP*) and grew in a polar manner (indicating homozygosity for *pLat52-PiRhoGDI1*) (Figure 3E,F).

**Figure 4:**
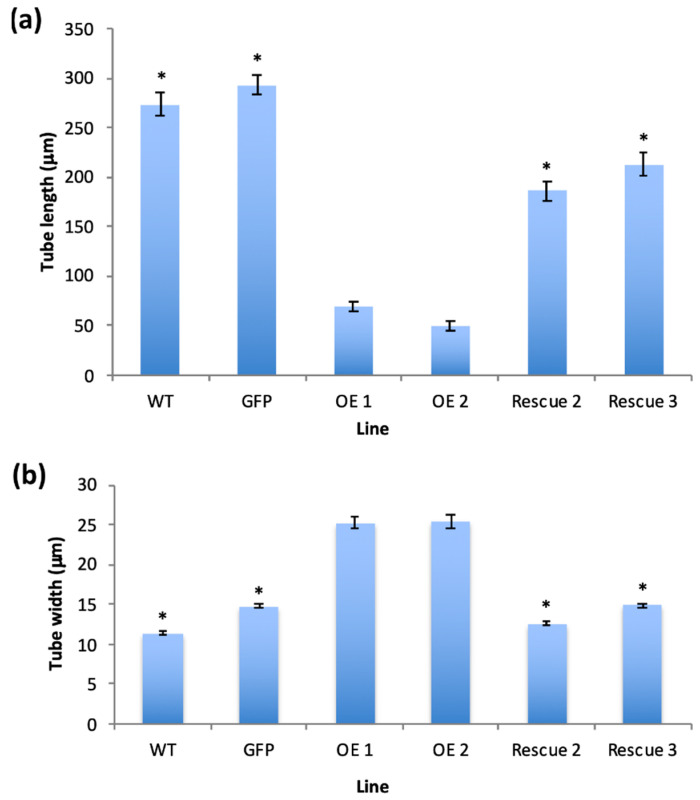
(**a**) Average pollen tube length (µm) and (**b**) average pollen tube width of the transgenic lines (as indicated) after 4 h of incubation at 28–30 °C. Plant lines shown: WT (wild type), GFP (transformant expressing GFP alone), OE1 and OE2 (transgenic lines expressing PiCDPK1-GFP alone), Rescue 2 and Rescue 3 (transgenic lines expressing PiCDPK1-GFP and PiRhoGDI1). Data are presented as means ± SE collected from >100 pollen tubes of each line, asterisks indicate significant differences between the samples labelled and the OE lines (Student’s *t*-test, *p* < 0.001).

Pollen from the various homozygous lines was cultured in vitro for 4 h and the phenotypes assessed. Pollen germination was not significantly different from wild type for any of the transgenic lines (Figure S3), in contrast there were significant differences in pollen tube phenotypes. Pollen expressing GFP alone grew with normal polarity to an average length of 293 µm, 107% of the average wild-type tube length (273.4 µm), the difference between the two was not statistically significant (Student’s *t*-test, *p* > 0.1) (Figure 3G,H). In contrast, consistent with previous transient expression studies, overexpression of PiCDPK1:GFP (OE lines) resulted in the majority of the pollen growing short, often almost spherical, pollen tubes (Figure 3G,H) [10]. Pollen from two independent lines over-expressing PiCDPK1:GFP (OE1 and OE2) alone grew to an average tube length of 68.6 µm (OE1) and 49.7 µm (OE2), 25% and 18%, respectively, of the average wild-type tube length (273.4 µm), both lines being significantly decreased in length relative to wild-type (Student’s *t*-test, *p* < 0.001). In contrast, lines expressing PiCDPK1:GFP and PiRhoGDI1 (Rescue lines) grew to an average length of 185.9 µm (Rescue 2) and 213 µm (Rescue 3), 68% and 78% respectively, of wild-type (Figure 4a). Both rescue lines exhibited significantly increased tube length compared with the overexpression lines (Student’s *t*-test, *p* < 0.001, Figure 3E,F and Figure 4a). Consistent with reduced length resulting from loss-of-polarity, tube width was also affected. Pollen expressing PiCDPK1:GFP alone displayed an average tube width of 25.3 µm (OE1) and 25.4 µm (OE2), 222% and 224% of and significantly different from wild type (11.4 µm) (Student’s *t*-test, *p* < 0.001) (Figure 4b). In contrast, rescue lines overexpressing both PiCDPK1:GFP and PiRhoGDI1 pollen grew to average widths of 14.9 µm (Rescue 3) and 12.6 µm (Rescue 2) these widths being significantly decreased relative to the OE lines (Student’s *t*-test, *p* < 0.001). Average pollen tube widths from rescue lines were more similar to those of the wild-type tubes, being 111% (OE1) and 131% (IE2) of the average wild-type tube width (Figure 3E,F and Figure 4b).

**Figure 5:**
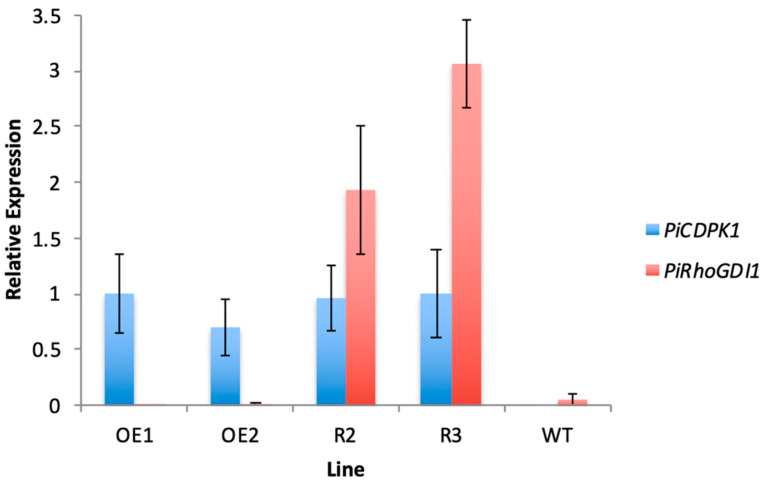
Quantitative PCR results showing relative expression of the PiRhoGDI transgene compared with the PiCDPK1 transgene in pollen from stable transgenic tobacco lines. Plant lines shown: OE1 and OE2 (transgenic lines expressing PiCDPK1-GFP alone), Rescue 2 and Rescue 3 (transgenic lines expressing PiCDPK1-GFP and PiRhoGDI1) and WT (wild type). Results are normalized to the ribosomal protein 25 (L25) internal reference gene and the OE1 line is used as the control (expression level of 1.0). Data are presented as means ± SE from three biological replicates.

In this experiment, both transgenes were driven by the pollen-specific promoter *pLat52*. This provided an advantage in that it increased the likelihood of expressing them at similar levels, but raised the possibility that addition of the second transgene could negatively affect the expression of the first. As a result, it was important to verify that phenotypic rescue was not trivially a result of a reduction in PiCDPK1 expression. To this end, qRT-PCR primers were designed to amplify a region spanning the junction of *pLat52* and the 5′ end of the PiCDPK1 or PiRhoGDI1 coding sequences. This minimized the potential of endogenous tobacco transcripts, which share high levels of identity with the transgenes, interfering with assessment of transgene expression. qRT-PCR was then used to assess expression levels of the two transgenes. Expression was normalized to internal *N. tabacum* reference genes, elongation factor 1-alpha (EF1) and ribosomal protein 25 (L25) [38]. As shown in Figure 5, the results of qRT-PCR indicated that expression levels of PiCDPK1 were very similar in the rescue and OE lines. Both rescue lines displayed similar expression of the PiCDPK1 transgene to the OE lines, and there was no significant difference between lines (Student’s *t*-test, *p* > 0.1) (Figure 5). No significant qRT-PCR signals were found in wild-type samples, supporting the idea that transgene expression was being measured specifically and endogenous RhoGDI and CDPK1 genes were not interfering with the assays. Overall, these results suggest that expression of PiRhoGDI1 at least partially rescues the effects of over-expression of PiCDPK1, and supports the hypothesis that PiCDPK1 possesses RhoGDF activity in vivo.

## 3. Discussion

**Figure 6:**
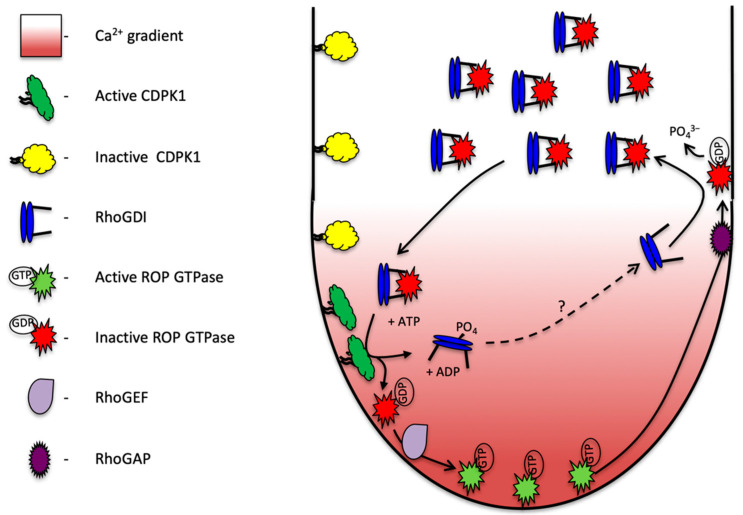
Proposed model of PiCDPK1 regulation of ROP in growing pollen tubes. Calcium-dependent protein kinase (PiCDPK1) is localized to the plasma membrane of the pollen tube. In the cytosol in the tube shank, Rho Guanine Dissociation Inhibitor (RhoGDI) binds to ROP GTPase removing to from the plasma membrane and locking it in an inactive state. At the tube tip, elevated calcium levels activate PiCDPK1, which phosphorylates the cytosolic RhoGDI:ROP GTPase complex. Upon phosphorylation ROP GTPase is released from the protein complex and recruited to the membrane where it is available for biological activation by Rho Guanine Exchange Factor (RhoGEF). Active (GTP bound) ROP GTPase interacts with and mediates the activity of downstream effectors to coordinate growth. As the tube extends, active ROP GTPase is passed back in the membrane until it interacts with ROP GTPase Activating Protein (RhoGAP), activating the enzyme activity of ROP causing it to hydrolyze GTP to GDP and become biologically inactivated. GDP bound ROP can be removed from the membrane by non-phosphorylated RhoGDI and recycled back to the tube tip. The dotted line and question mark indicate the activity of an as yet unidentified phosphatase the activity of which is needed to re-activate RhoGDI.

The data presented provide several lines of evidence that PiRhoGDI1 interacts with, and is likely a substrate of, the calmodulin-domain protein kinase PiCDPK1. Though the regulatory site(s) were not identified, PiCDPK1 interacted with and phosphorylated PiRhoGDI1 in vitro. The ability of PiRhoGDI1 to rescue the effect of PiCDPK1 in pollen tubes provides support for this interaction occurring in vivo. The homology based predicted activity of PiRhoGDI1 to bind ROP GTPase was confirmed using the yeast 2-hybrid assay. As RhoGDI is an established regulator of ROP GTPase this suggests that the pathway leading to the PiCDPK1 loss of polarity phenotype involves the activity of ROP GTPase. Combined these results are consistent with PiCDPK1 regulating PiRhoGDI1 by phosphorylation, and that this phosphorylation mediates the ability of PiRhoGDI1 to bind ROP GTPase. The observed phenotypes are consistent with the scenario that when PiCDPK1 alone is over-expressed, the negative regulatory activity of endogenous RhoGDI is neutralized by excess kinase activity, leading to increased recruitment of ROP to the plasma membrane. Co-over expressing PiRhoGDI with PiCDPK1, titrates much of the excess kinase activity away from endogenous RhoGDI reducing ROP membrane recruitment and resulting in a more polar growth phenotype. Extrapolating from these results, and integrating them with the current knowledge of the functioning of RhoGDI’s and the regulation of ROP GTPases and Ca^2+^ in tip growth, allows us to propose a model for the integration of these components into a pathway which mediates rapid recycling and regulation of ROP at the pollen tube tip (Figure 6).

In this model, ROP GTPase is held inactive in a cytosolic complex with PiRhoGDI1. Under high cytosolic Ca^2+^ concentrations at the pollen tube tip, PiCDPK1, which is localized to the plasma membrane [10], is activated to phosphorylate PiRhoGDI1. This phosphorylation leads to release of ROP from the complex, exposure of its hydrophobic prenyl side chain and plasma membrane recruitment. At the plasma membrane, ROP is stimulated to exchange GDP for GTP by a GEF and biologically activated to promote polarized growth. As the tube tip extends and secretory vesicles are deposited, biologically active GTPase is progressively carried in a posterior direction by membrane flow. GTPase activating proteins (GAPs) are absent at the tube tip, but present in the plasma membrane in the tube shank (their recruitment to this localization being mediated by a 14:3:3 protein and possibly phosphorylation by phosphoglycerate kinase [39,40]). In the tube shank, where PiCDPK1 is inactive due to lower cytosolic Ca^2+^ concentration, GAPs stimulate the GTPases to hydrolyze GTP to GDP leading to biological inactivation. Non-phosphorylated RhoGDI can now extract the GDP-bound GTPase from the plasma membrane and recycle the GTPase back to the pollen tube tip. As a whole, this pathway facilitates rapid recycling of ROP GTPase to the growing tip, the role of PiCDPK1 being that of a GDF.

In this pathway, Ca^2+^ influx at the growing pollen tube tip provides the spatial information that leads to the plasma membrane recruitment, placing it in position for subsequent activation of ROP. This model is consistent with many previously reported observations. First, though the majority of ROP in pollen tubes is cytosolic, the active fraction localizes to the apical region of the plasma membrane, coincident with the peak of the tip focused Ca^2+^ gradient [17]. This tip region is the only location in pollen tubes that has cytosolic Ca^2+^ levels sufficient for PiCDPK1 activation [10]. As noted above, ROP has been reported to be upstream of cytosolic Ca^2+^ through a pathway involving RIC3 [41]. This being the case, Ca^2+^ and PiCDPK1 act downstream of ROP in addition to regulating its activation and these molecules thus form a positive feedback loop (Figure 6). Such feedback loops are common components of GTPase pathways involved in polarity and exist in pollen tubes [1,10,42,43]. Empirical support for involvement of PiCDPK1 in such a feedback loop is provided by its over-expression not only leading to loss of growth polarity but also dramatic elevation of cytosolic Ca^2+^ within the tube tip [10]. The model proposed is also consistent with a report that mutant ROP, lacking the ability to interact with RhoGDI, accumulates at the plasma membrane in the pollen tube shank and does not exhibit the tip localization of wild type ROP [39]. Lastly, more than 25 years ago it was shown that asymmetric release of caged Ca^2+^ within a pollen tube tip leads to growth re-orientation, focused to the location of Ca^2+^ release [44]. The model proposed provides a mechanism to explain how this phenomenon occurs.

The signaling pathway proposed facilitates the perpetuation of rapid recruitment of ROP to the plasma membrane at the tip of pollen tubes. Currently missing from this model are potential mechanisms for negative regulation of the feedback loop. One likely mechanism is the dephosphorylation of PiRhoGDI1 by a protein phosphatase behind the tip, reactivating its ability to extract ROP from the plasma membrane (illustrated in Figure 6). In human cancer cells, dephosphorylation of RhoGDI occurs through the activity of a PPM family phosphatase, protein phosphatase 1b (PPM1B), which acts antagonistically and results in suppressed Rho GTPase activity [45]. Consistent with dephosphorylation of RhoGDI playing a similar role in pollen tubes, type 2A protein phosphatase inhibitors induce a phenotype similar to ROP over-expression [46]. Another significant question not included in the model proposed is the nature of mechanism(s) by which polarity is initially established at pollen germination. Establishment of polarity may require additional signaling events, but the simplest scenario does not. Pollen plasma membranes possess stretch-activated Ca^2+^ channels [47,48,49,50]. On the stigma surface, stress is likely to be focused on the direction of water uptake, and as a result maximal activation of Ca^2+^ channels may occur proximal to the point of attachment on the stigma. A Ca^2+^ gradient created in this fashion could potentially activate the feedback loop outlined in Figure 6. Support for this scenario comes from the observation that pollen grains usually germinate from the pollen wall aperture closest to the stigma surface, but in very high humidity they will sometimes germinate in random directions [51]. For simplicity, we omitted the regulation of GEF activation of ROP from this model, but considerable progress has been made in this area. RhoGEF localization in pollen tubes has been shown to be mediated by their phosphorylation by AGC kinases, phosphorylation of the PRONE domain of RhoGEF leading to localization to the apical tip of the pollen tube and loss of this interaction causing ectopic targeting of RhoGEF [23]. Furthermore, tomato RopGEF/KPP interacts with the kinase domain of pollen-specific receptor-like kinases LePRK1 and LePRK2 [31] and studies in *Arabidopsis* have demonstrated that interaction between PRK and RhoGEF results in activation of ROP GTPase [52]. Excitingly, as these receptor-like kinases possess extracellular domains, these interactions provide a mechanism by which ROP GTPase activation can be mediated by external ligands providing a mechanism for the chemotropism exhibited by pollen tubes. Interestingly PiCDPK1 might itself play a direct role in regulating ROP it was recently reported that its’ *A. thaliana* homologs CPK17 and -34, phosphorylates At ROP1 at S97, though whether this phosphorylation has any biological relevance is currently unclear [53].

Regulation of RhoGDI by a CDPK isoform has significant implications to our understanding of the regulation of growth polarity in plant cells in general. ROP isoforms have been implicated in polarity in a variety of plant cell types [54], and RhoGDI mutants exhibit defective polar growth in root hairs in *Arabidopsis* [55] hence it is likely that similar pathways employing isoforms of these enzymes are involved in many cell types. For example, a RhoGDI was shown to be critical for nuclear migration in emerging root hair cells [56], and was demonstrated to act in tandem with vesicular trafficking to regulate Rop GTPase signaling in leaf pavement cells [57]. In a broader context, fluctuations of cytosolic Ca^2+^ are associated with most aspects of growth and development, and responses to environmental signals in plants [58,59,60,61]. The majority of Ca^2+^-stimulated kinase activity in plant cells appears to be associated with CDPKs and these molecules are thought to be pivotal to many Ca^2+^ regulated processes [62,63,64,65,66]. Though a considerable number of CDPK isoforms have been identified from multiple plant species, only some have clearly defined biological functions [58,67]. Many processes involving GTPase signaling are associated with Ca^2+^ transients and Ca^2+^-regulated kinase activity [54,58], and we speculate that CDPK-mediated membrane recruitment of ROP GTPases is likely to be significant to a wide range of cellular processes in plant cells.

## 4. Materials and Methods

### 4.1. Yeast 2-Hybrid Library Screening

A pGBD/∆N-PiCDPK1 bait plasmid was constructed by amplifying PiCDPK1 cDNA clone (Genbank acc. # DQ147913) by PCR using primers: ∆N-PiCDPK1-F, 5′-GGATCCAGGGGCCAACCTAAT-3′ and PiCDPK1-K 5′-GTCGACCTCCTTGATCCAAGG-3′. The amplicon was cloned into the *Bam* H1/*Sal* I sites of pGBD-C1 (20). Yeast strain AH109 was sequentially transformed with pGBD/∆N-PiCDPK1 and a pollen cDNA library in pGAD424 by the LiAc method. In the first phase of selection (-his) transformants were plated onto synthetic dropout (SD) medium lacking leu, tryp, and his. After 7 days, cells were replica-plated onto SD medium lacking leu, tryp, his, and ade. Plasmid DNA was extracted from positive clones, transformed into *E. coli* and plated on LB agar supplemented with 100 µg/mL ampicillin. Plasmid DNA extracted from *E. coli* was back-transformed into yeast strain AH109 with the bait and cultured on SD medium lacking leu, tryp, his to confirm interactions. To generate an in-frame PiRhoGDI1 bait construct, PiRhoGDI1 was amplified with primers PiRhoGDI1-BH1-F (5′-GGATCCATGTCAGCTATTGTTGA-3′) and PiRhoGDI1-Sal1-R (5′-GTCGACTCAGAGCTGAAGCCA), to add 5′ *Bam* HI and 3′ *Sal* I sites. The amplicon was cloned into the *Bam* HI/*Sal* I sites of pGAD-C1 [68].

### 4.2. Expression of Recombinant Proteins

pRSET-B vector (Invitrogen, Waltham, MA, USA) was used to express full-length PiCDPK1 and PiRhoGDI1 6X N-terminal His-fusion proteins. Expression of PiCDPK1 was performed as previously reported [10]. The PiRhoGDI1 expression construct was generated by PCR by using PiRhoGDI1-NcoI-5′ (5′-CCATGGTAATGTCAGCTATTGTTGAAC-3′) and PiRhoGDI1-NcoI-3′ (5′-CCATGGCCCAGAGCTGAAGCCA-3′). The amplified fragment was verified by DNA sequencing and cloned into the Nco I site of pRSET-B. Expression and purification of His-tagged-PiRhoGDI1 were performed as previously described [10].

### 4.3. Pull-Down Assay

Pull-down assays were performed by mixing 2 µg of each 6X His-tag fusion protein with phenyl sepharose beads in 500 µL of binding buffer (20 mM HEPES, pH 7.5, 5 mM MgCl_2_, 1 mM DTT, and 0.1% Triton X-100) with 1 mM Ca^2+^. Samples were rotated for 2 h at 4 °C, pelleted, and washed three times with wash buffer (binding buffer supplemented with 0.3 M NaCl). Proteins were eluted in 1X SDS sample buffer, resolved on a 12% SDS-PAGE gel and transferred to Immobilon-P membrane (Millipore, Bedford, MA, USA). Fusion proteins were detected using a monoclonal Anti-polyHISTIDINE Clone HIS-1 antibody (Sigma-Aldrich, St Louis, MO, USA), anti-mouse IgG alkaline phosphatase conjugated 2° antibody (Sigma, St. Louis, MO, USA) and WesternBlue stabilized alkaline phosphatase substrate (BioRad, La Jolla, CA, USA) all following the manufacturers’ recommended protocols.

### 4.4. Phosphorylation Assay

A total of 0.6 µg PiCDPK1-6X His-tag fusion protein was incubated with 0.5 µg PiRhoGDI1-6X His-tag in phosphorylation buffer (50 mM HEPES, pH 7.0, 1 mM MgCl_2_, 1 mM DTT) with either 50 µM Ca^2+^ or 1 mM EGTA. Reactions were initiated by addition of 10µCi [γ-^32^P] ATP, incubated for 10 min at room temperature then terminated by adding 5X SDS sample buffer. Samples were electrophoresed on 10% SDS-PAGE gels, blotted to Immobilon-P membrane and exposed to autoradiography.

### 4.5. Stable Expression in Tobacco

The previously constructed *pLat52-PiCDPK1:GFP* construct [10] subcloned from pBluescript into the plant transformation vector pBI101. This construct was used to generate transgenic *Nicotiana tabacum* using *Agrobacterium*-mediated plant transformation. We generated homozygous lines by identifying transgenic lines displaying GFP fluorescence as well as the PiCDPK1 overexpression phenotype of ballooning pollen tubes. These were self-fertilized and the resultant seed used to generate progeny. Once flowering, the pollen phenotypes of the progeny assess to identify lines with 100% of pollen tubes displaying GFP fluorescence and the PiCDPK1 overexpression phenotype (homozygous lines). A line was selected for re-transformation. For the second transformation we used the plant transformation vector pGreenII 0179 (kindly provided by Dr. Henning Kunz, SBS WSU) to transform *Agrobacterium* strain GV3101 with our gene of interest coding for PiRhoGDI1 driven by the pollen-specific *Lat52* promoter. The common sequencing primers M13 Forward and M13 Reverse were used to amplify the Lat52:PiRhoGDI1:Nos transgene cassette from the pBluescript vector as previously built [10]. PCR reactions were resolved on an agarose gel, excised and recovered using the ZymoClean DNA Gel Recovery Kit according to the manufacturers protocol. DNA fragments were digested with restriction enzymes and subsequently ligated into pGreenII. We then re-transformed the homozygous *N. tabacum* overexpressing PiCDPK1:GFP with the GV3101 *Agrobacterium* containing our PiRhoGDI1 construct and regenerated the plants using tissue culture methods. Plants were screened by RT-PCR to verify expression of PiRhoGDI1 and pollen tube phenotypes assessed. Once plants possessing both transgenes were identified, they were self-pollinated to generate lines homozygous for both transgenes.

### 4.6. Analysis of Transformed Pollen Tubes

Confocal images were taken with a Leica TCS SP8 confocal laser-scanning microscope (Leica Microsystems, Wetzlar, Germany) at the 514-nm excitation wavelength to image GFP fluorescence. Light micrographs were also taken for each sample and the pollen tubes quantitatively analyzed by measuring tube lengths with the FIJI distribution of ImageJ [69].

### 4.7. Quantitative RT-PCR

RT-qPCR was performed at the WSU Genomics Core on an ABI 7500 Fast thermocycler (ABI, Vernon, CA, USA) with the ability to perform real time quantification of PCR products. RNA was extracted from pollen using the Trizol reagent (Invitrogen, Carlsbad, CA, USA) according to the manufacturers protocol. cDNA was generated using the SensiFAST cDNA Synthesis Kit (BioLine, London, UK) which was used as the template DNA to perform qPCR using the SensiFAST SYBR Lo-ROX Kit (BioLine, London, UK), both according to the manufacturers’ protocol. The forward primer for quantifying both the PiCDPK1 and PiRhoGDI1 transgenes was designed to span the 3′ end of the Lat52 promoter coding sequence: Lat52 F (5′-CACACACAAAGAGAAGGAGCA-3′). Reverse primers were designed to span the 5′ end of the PiCDPK1 coding sequence (5′-GCAGTGCTGATGGATTCTG-3′) and the 5′ end of the PiRhoGDI1 coding sequence (5′-CCTCTTCTTGCTCAATTCCA-3′). Control primers used for normalization were designed to amplify a small 215bp fragment of the tobacco *L25* coding sequence: L25F (5′-CAAGGCTGTCAAGTCAGGA-3′), L25R (5′-AGGGTGTTGTTGTCCTCAATC-3′).

## Data Availability

The data presented in this study are available within the article or Supplementary Materials.

## Supplementary Materials

The following supporting information can be downloaded at: https://www.mdpi.com/article/10.3390/plants11030254/s1, Figure S1, RNA blots showing the developmental profile of PiRhoGDI expression, Figure S2, yeast 2-hybrid assay of PiRhoGDI1 and PiRop1 interaction.
