# Supplementary material for: Calmodulin Domain Protein Kinase PiCDPK1 Regulates Pollen Tube Growth Polarity through Interaction with RhoGDI
Source: Plants (Basel). 2022 Jan 19;11(3):254. doi: 10.3390/plants11030254 (PMC8838988; doi:10.3390/plants11030254)
Supplement: Supplementary file 1 [file plants-11-00254-s001.zip › plants-1558139-supplementary.pdf]

plants MDPI

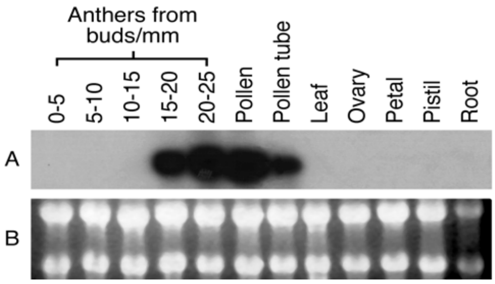

Supplemental figure 1. Expression pattern of PiRhoGDI1

(**A**) Autoradiograph of an RNA gel blot representing various tissues (as labeled), hybridized with PiRhoGDI1 cDNA. (**B**) Ethidium bromide staining of the gel used to generate the blot shown in (A), illustrating RNA loading (15 µg total RNA/lane). PiRhoGDI1 expression is first detectable in anthers 15-20 mm buds, rises to a peak in mature pollen and remains high in pollen tubes. Expression was not detected in other tissues.

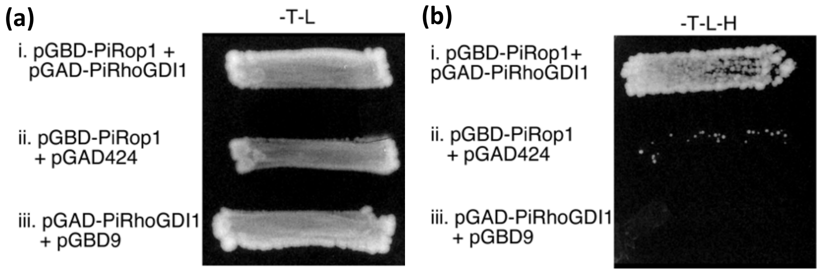

Supplemental figure 2. PiRhoGDI1 interacts with PiRop1 in a yeast 2-hybrid assay Yeast transformed with bait (pGBD) and prey (pGAD) constructs or empty vectors (as labeled), were grown on (**a**) media lacking tryptophan and leucine (plasmid selection), and (**b**) media lacking trytophan, leucine and histidine, providing selection for both plasmids and interaction of fusion proteins). Only cells transformed with pGBD-PiCDPK1 and pGAD-PiRhoGDI1 were able to well on histidine selection, suggesting that the cDNAs encoded by these clones encode proteins that interact.

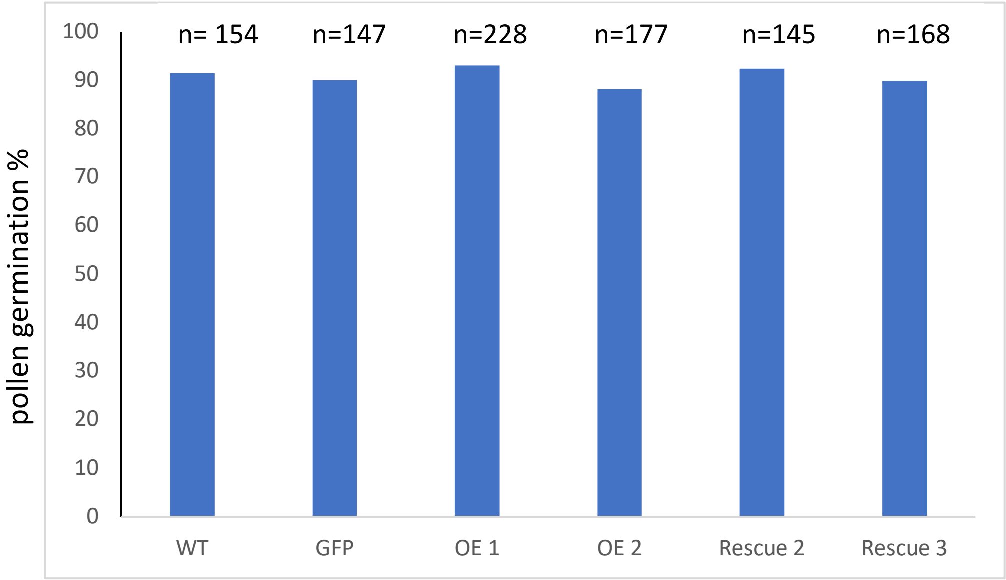

Supplemental figure 3. In vitro pollen germination rates (%) of wild type and transgenic lines (as indicated). N = number of pollen grains scored for each sample. Plant lines shown: WT (wild type), GFP (transformant expressing GFP alone), OE1 and OE2 (transgenic lines expressing PiCDPK1-GFP alone), Rescue 2 and Rescue 3 (transgenic lines expressing PiCDPK1-GFP and PiRhoGDI1).
